# Effectiveness of Traditional Chinese Exercise for Symptoms of Knee Osteoarthritis: A Systematic Review and Meta-Analysis of Randomized Controlled Trials

**DOI:** 10.3390/ijerph17217873

**Published:** 2020-10-27

**Authors:** Ruojin Li, Hongwei Chen, Jiahao Feng, Ying Xiao, Haoyang Zhang, Christopher Wai-Kei Lam, Hong Xiao

**Affiliations:** 1 Department of Physical Education, Sun Yat-Sen University, Guangzhou 510000, China; lirj33@mail2.sysu.edu.cn; 2 Faculty of Medicine, Macau University of Science and Technology, Taipa, Macau 999078, China; musthwchen@gmail.com (H.C.); yxiao@must.edu.mo (Y.X.); 3 State Key Laboratory of Quality Research in Chinese Medicines, Macau University of Science and Technology, Avenida Wai Long, Taipa, Macau 999078, China; 4 School of Public Health (Shenzhen), Sun Yat-Sen University, Shenzhen 518000, China; 5 School of Medicine, Sun Yat-Sen University, Guangzhou 510000, China; fengjh27@mail2.sysu.edu.cn; 6 School of Data and Computer Science, Sun Yat-Sen University, Guangzhou 510000, China; zhanghaoyang0@hotmail.com

**Keywords:** traditional chinese exercise, tai chi, baduanjin, knee osteoarthritis (KOA), systematic review, meta-analysis

## Abstract

*Background:* Growing evidences have advocated the potential benefits of traditional Chinese exercise (TCE) on symptomatic improvement of knee osteoarthritis (KOA). However, most of them have been derived from cross-sectional studies or case reports; the effectiveness of TCE therapies has not been fully assessed with a randomized control trial (RCT). In order to evaluate the combined clinical effectiveness of TCE for KOA, we conducted a systematic review and meta-analysis on the existing RCTs on KOA. *Methods:* A systematic search was performed in four electronic databases: PubMed, Web of Science, Cochrane Library, and EMBASE from the time of their inception to February 2020. All eligible RCTs were included in which TCE was utilized for treating KOA as compared to a control group. Two reviewers independently extracted the data and evaluated the risk of bias following the Cochrane Risk of Bias Tool for RCT. The symptoms of KOA evaluated by the Western Ontario and McMaster Universities Arthritis Index (WOMAC) and the Knee Injury and Osteoarthritis Outcome Score (KOOS) were regarded as the primary outcomes in this study. Each outcome measure was pooled by a standardized mean difference (SMD) with 95% confidence intervals (CI). A meta-analysis was applied with a random or fixed effect model for the collected data to calculate the summary SMD with 95% CI based on different statistical heterogeneity. In addition, subgroup analyses were used to investigate heterogeneity and sensitivity analysis was carried out for the results of the meta-analysis. Egger’s test and the funnel plots were used to examine the potential bias in the RCTs. *Results:* A total of 14 RCTs involving 815 patients with KOA were included. Compared with a control group; the synthesized data of TCE showed a significant improvement in WOMAC/KOOS pain score (SMD = −0.61; 95% CI: −0.86 to −0.37; *p* < 0.001), stiffness score (SMD = −0.75; 95% CI: −1.09 to −0.41; *p* < 0.001), and physical function score (SMD = −0.67; 95% CI: −0.82 to −0.53; *p* < 0.001). Conclusions: Our meta-analysis suggested that TCE may be effective in alleviating pain; relieving stiffness and improving the physical function for patients with KOA. Yet; given the methodological limitations of included RCTs in this meta-analysis; more high-quality RCTs with large sample size and long-term intervention are required to further confirm the effectiveness and underlying mechanisms of TCE for treating KOA.

## 1. Introduction

Knee osteoarthritis (KOA) is a kind of degenerative knee-joint disease, which is also a leading cause of disability accounting for 9.6% men and 18.0% women aged over 60 years [1]. As a degenerative and progressive knee-joint disease, the development of KOA is closely associated with multiple factors, including demographic factors such as age, over-weight, and sex, and sport injuries factors such as muscle weakness, joint laxity, and bone trauma [2]. Since KOA causes disability and impacts on the patient’s quality of life, it has become a major public health issue worldwide [3]. In addition to the surgical treatment involving joint reconstruction, other treatments of KOA can be broadly categorized as pharmacological and non-pharmacological [4,5]. Although the pharmacological treatment can be effective for reducing pain and improving physical function in KOA [6,7,8], more and more research studies have shown that the long-term usage of medicines and intra-articular injection may cause adverse effects such as gastrointestinal reaction, multi-organ failure, pain, and swelling [9,10,11,12]. Moreover, surgery and a long-term pharmacological treatment may cause a great impact on society and individuals with enormous health-care expenditures and reduction in the quality of life [13,14].

In contrast, non-pharmacological interventions including physical, psychological, and mind-body exercises are relatively safe and effective [15]. They have been strongly recommended by the Osteoarthritis Research Society International (OARSI), the American College of Rheumatology (ACR), and the American Academy of Orthopedic Surgeons (AAOS) [4,16,17]. Overall, a non-pharmacological intervention may be an important option for either the society or patients.

Traditional Chinese exercise (TCE), as a therapeutic, aerobic, and mind-body exercise originated from traditional Chinese medicine tracing back to approximately three thousand years ago [18,19,20]. TCE, as a major integral part of non-pharmacological intervention, includes Tai Chi, Baduanjin, Yijinjing, and Wuqinxi that are characterized by slow, gentle, and symmetrical movements, musculoskeletal stretching, physical and psychological relaxation, combined with deep diaphragmatic breathing [21,22,23,24]. Increasing research and practices have demonstrated that TCE is effectively beneficial for improving physical status and modulating the psychological health of patients with mental disorders [21], metabolic syndrome [24], Parkinson’s disease [25], post-myocardial infarction [26], chronic obstructive pulmonary disease [27], type 2 diabetes mellitus [28], chronic pain disease [29], and cancer [30]. TCE is also beneficial for limb rehabilitation which includes limb motor function, balance function, daily life activities, and neurological improvement [31].

Currently, increasing numbers of clinical trials and meta-analyses have reported that TCE has been used for treating KOA [18,22,32,33,34,35]. More and more RCTs have indicated that TCE can significantly improve the symptoms of KOA including pain reduction, relief of stiffness, and improvement on physical function [33,34,35]. However, results of different trials are inconsistent, with several trials suggesting that TCE had no effects on those outcomes probably due to the small sample size, short duration time, and the severity of KOA in participants [36,37]. The conclusions from current studies have remained controversial. Moreover, a previous systematic review and meta-analysis has demonstrated that TCE can significantly improve symptoms of KOA, but it has insufficient subgroup analysis and included ineligible studies [18]. Therefore, it is appropriate to further investigate the effectiveness of TCE for patients with KOA, aiming to help doctors and other health-care professionals plan stage-specific treatment for patients.

## 2. Methods

This systematic review and meta-analysis was reported in accordance with the Preferred Reporting Items for Systematic Reviews and Meta-Analyses (PRISMA) guidelines [38].

### 2.1. Search Strategy

A literature search was conducted by two independent researchers (R.L. and H.C.). The systematic search of data was performed in four electronic databases: PubMed, Web of Science, Cochrane Library, and EMBASE to identify effects of TCE on symptoms of KOA. We included papers published from the time of their inception to February 2020 in English with the following keywords: {(Traditional Chinese exercise [Title/Abstract] OR “Tai Chi” [Title/Abstract] OR “Qigong” [Title/Abstract] OR Yijinjing [Title/Abstract] OR Baduanjin [Title/Abstract] OR Wuqinxi” [Title/Abstract]) AND (“knee osteoarthritis” [Title/Abstract] OR “knee arthritis” [Title/Abstract] OR gonitis [Title/Abstract] OR gonarthritis [Title/Abstract]) AND (“randomized controlled trial” [Title/Abstract] OR randomization [Title/Abstract] OR randomized [Title/Abstract])}. A more detailed search strategy is in the supplementary material (Table S1).

### 2.2. Inclusion and Exclusion Criteria

Articles were included for this research based on the following criteria: (i) The study design was a randomized controlled trial (RCT); (ii) participants were diagnosed with KOA by validated criteria, such as those of the American College of Rheumatology (ACR), the American Rheumatism Association (ARA), the Kellgren Lawrence classification (KL), radio-graphic evidence or physician-confirmed diagnosis [39,40,41]; (iii) outcome measures included the assessment of pain intensity, stiffness, and physical function for KOA using the Western Ontario and McMaster Universities Osteoarthritis Index (WOMAC), and the Knee Injury and Osteoarthritis Outcome Score (KOOS) [42,43]; (iv) the trial explored the efficacy of different traditional Chinese exercises, as compared with a control or comparison group (e.g., physical therapy, health education, sham exercise) on symptoms of KOA.

Articles were excluded based on the following criteria: (i) Those that were study-designed (not RCT); (ii) participants also had hip osteoarthritis; and (iii) the article was retracted.

### 2.3. Study Selection

After a systematic search, the retrieved articles were screened for eligibility based on the above inclusion and exclusion criteria by two independent reviewers (R.L. and H.C.) to confirm that the title and abstract conformed with the criteria. Then, the potentially eligible articles were further read in full text for assessment. Discrepancies were resolved by the third reviewer (J.F.).

### 2.4. Data Extraction

Three reviewers (R.L., H.C., and J.F.) independently rated the included paper and extracted the data. Details of the retrieved articles are summarized in Table 1. The following data were extracted from the retrieved articles: (i) Reference (first author and year of publication); (ii) study location; (iii) characteristics of participants; (iv) intervention protocol; (v) outcomes measure; and (vi) adverse effects [44].

### 2.5. Risk of Bias Assessment in Individual Trials

Two reviewers (R.L. and H.C.) independently evaluated the risk of bias using the following criteria adopted from the Cochrane Risk of Bias Tool for Randomized Controlled Trials: Selection bias, performance bias, attribution bias, reporting bias, and other bias. The classification of risk of bias was divided into “Low risk of bias”, “High risk of bias”, or “unclear” [45]. Disagreements were resolved by a third reviewer (J.F.). The extent of agreement between the reviewers was calculated using the Kappa coefficient (k = 0.908).

### 2.6. Statistical Analysis

All statistical analyses were performed using the Review Manager 5 software (version 5.3, The Nordic Cochrane Centre, Copenhagen, Denmark). The symptoms of KOA were evaluated using the Western Ontario and McMaster Universities Arthritis Index (WOMAC) and the Knee Injury and Osteoarthritis Outcome Score (KOOS), which were regarded as the primary outcomes in this research. We extracted the quantitative data from all selected RCTs including sample size, as well as the mean and standard deviation of outcome measures at baseline and post-intervention in each group. For continuous outcomes with different scoring units, the standardized mean difference (SMD) with 95% confidence intervals (CI) was used to pool each outcome measure for estimating the effect size. Statistical heterogeneity was evaluated using the I^2^ test to identify the difference in results, and was categorized as: (i) I^2^ ≤ 25%, low heterogeneity; (ii) 25% < I^2^ < 50%, moderate heterogeneity; (iii) 50% < I^2^ < 75%, substantial heterogeneity; (iv) I^2^ ≥ 75%, high heterogeneity. A fixed-effect model was applied to evaluate the summary SMD with 95% CI when I^2^ < 50% and *p* > 0.01; otherwise, a random-effect model was applied [46,47]. In addition, subgroup analyses were used to investigate the heterogeneity involving the exercise type (Tai Chi and Baduanjin), geographical location (Asian populations and non-Asian populations), duration time (8 and 12 weeks), sample size (no. of participants ≥ 30 and < 30), and control group type (active control group and passive control group). Sensitivity analysis was carried out for the results of the meta-analysis, which included the outcomes of pain, stiffness, and physical function using the WOMAC or KOOS score [46]. When the number of all included trials was ≥10, Egger’s test and the funnel plots were used to examine the potential bias in the RCTs included in this meta-analysis [45].

## 3. Results

### 3.1. Study Selection

**Figure 1:**
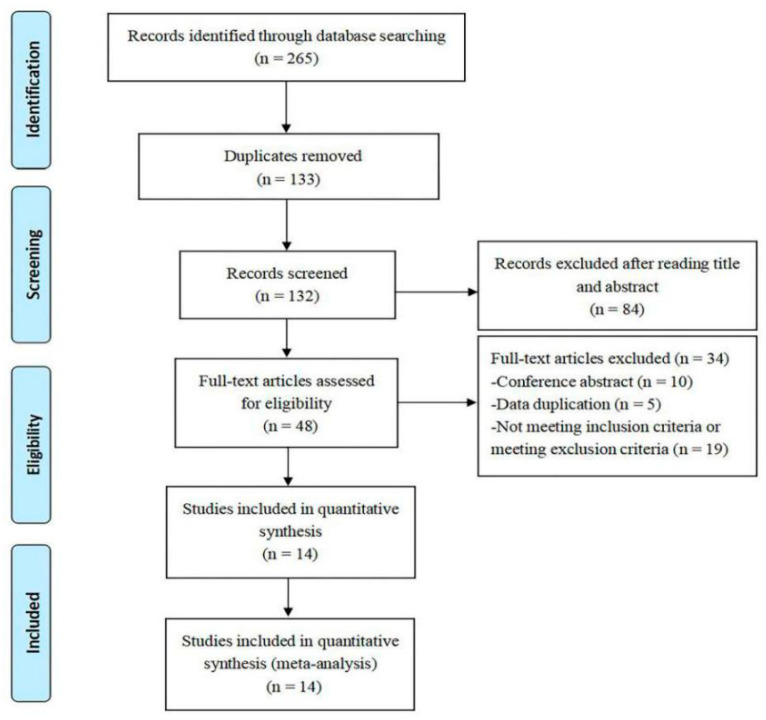
Flowchart of the studies selection process.

As shown in Figure 1, there were 265 articles identified through four different electronic database searches. Among them, 133 were duplicated and excluded. A total of 84 records of reviews, case reports, protocol, and commentary were excluded after screening the title and abstract. Then, full-texts of the remaining 48 articles were further assessed, and 34 full-text articles were excluded due to the following three reasons: Conference papers (*n* = 10); data duplication (*n* = 5); not meeting our inclusion criteria or meeting exclusion criteria (*n* = 19). Finally, a total of 14 RCTs were included in this meta-analysis.

### 3.2. Study Characteristics 

Characteristics of study location, participant data, intervention protocol, outcomes measured, and adverse effects from the assessed studies are summarized in Table 1.

Together, the 14 RCTs contained a total of 815 participants, and all were published in English before February 2020 [33,34,35,36,37,48,49,50,51,52,53,54,55,56]. The geographical location of the studies originated from China, Iran, USA, or Korea. Participants were diagnosed with KOA by the American College of Rheumatology classification criteria (ACR), the diagnostic criteria of the American Rheumatism Association (ARA), the Kellgren Lawrence classification (KL), radio-graphic evidence, or physician-confirmed.

For intervention programs, 11 studies applied the Tai Chi exercise [33,35,36,37,49,50,52,53,54,55,56] and two studies applied the Baduanjin exercise [34,48]. Only one study applied both the Tai Chi and Baduanjin exercise [51]. Of these studies, the duration time of interventions varied from 4 to 24 weeks. There were 12 studies that measured the symptoms of KOA using the Western Ontario and McMaster Universities Osteoarthritis Index (WOMAC). The WOMAC scale is a self-administered questionnaire consisting of 24 items divided into three sub-scales: Pain, stiffness, and physical function [42]. However, scoring of the WOMAC was reported differently among studies, with ten reporting on a scale of 0–100 [34,35,36,37,48,49,50,53,54,55] and two reporting on a scale score of 0–1700 [33,56], with higher scores reflecting a worse condition. In addition, two other studies used the Knee Injury and Osteoarthritis Outcome Score (KOOS) in order to measure the symptoms of KOA [51,52]. This is an extension of the WOMAC scale, which covers five patient-relevant dimensions: Pain, other disease-specific symptoms, activities of daily living function, sport and recreation function, and knee-related quality of life, with a score of zero representing extreme knee problems and a score of 100 representing no knee problems [43]. Finally, only one study reported adverse events and the remaining studies did not report any adverse events.

### 3.3. Assessment of Risk of Bias

**Figure 2:**
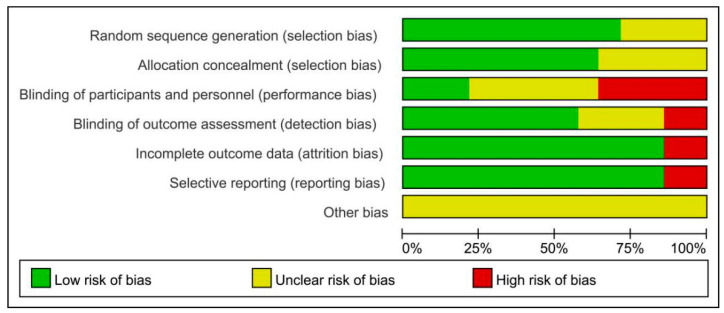
Risk of bias graph.

**Figure 3:**
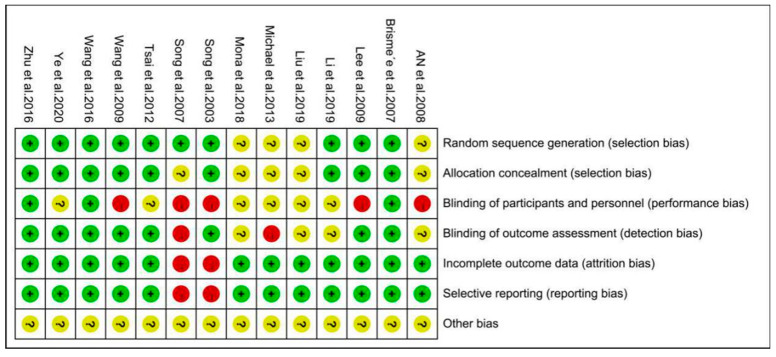
Risk of bias summary.

Results of the assessment of risk of bias for all the included studies are summarized in Figure 2 and Figure 3. With regard to the risk of selection bias, 10 of all the included studies in random sequence generation were at low risk [33,34,35,36,49,50,53,54,55,56] and nine of all the included studies in allocation concealment were at low risk [33,34,35,36,49,50,53,55,56], while the remaining studies were unclear mentioning only randomization without describing clearly their method of random sequence generation and allocation concealment, respectively [37,48,51,52,54]. Of these studies, only three studies were at low risk in the risk of performance bias [33,35,49]. For the risk of detection bias, eight studies were at low risk that adopted the blinding of outcome assessment [33,34,35,36,49,53,55,56]. Moreover, most of the studies were at low risk in the risk of attrition bias and reporting bias, only two studies were at high risk with no reported trial registration [53,54]. All the included studies were unclear in the risk of other bias.

### 3.4. Outcome Measures

**Figure 4:**
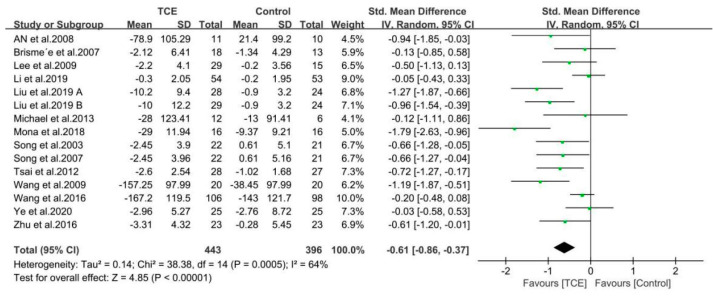
Forest plots showing standardized mean difference of change on the pain score (WOMAC/KOOS) between traditional Chinese exercise (TCE) group and a control/comparison group. CI: Confidence Interval; KOOS: The Knee Injury and Osteoarthritis Outcome Score; WOMAC: The Western Ontario and McMaster Universities Arthritis Index.

**Figure 5:**
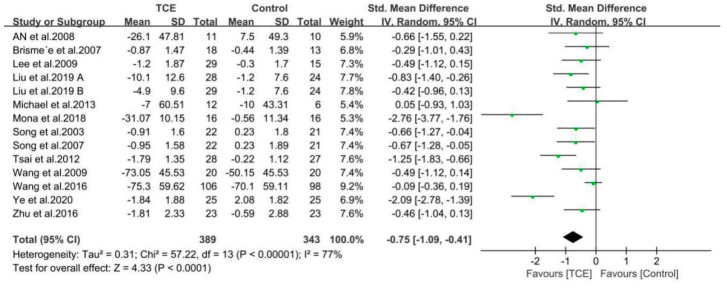
Forest plots showing standardized mean difference of change on the stiffness score (WOMAC/KOOS) between TCE group and a control/comparison group. CI: Confidence Interval; KOOS: The Knee Injury and Osteoarthritis Outcome Score; WOMAC: The Western Ontario and McMaster Universities Arthritis Index.

**Figure 6:**
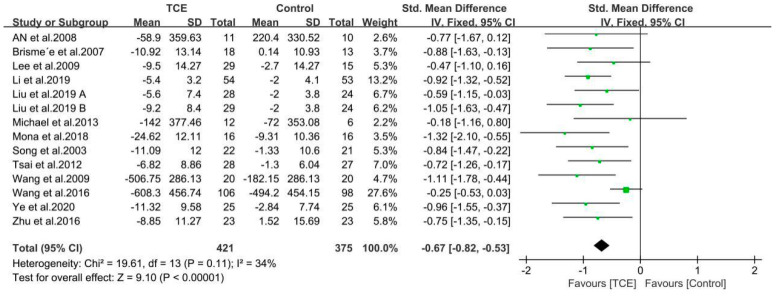
Forest plots showing standardized mean difference of change on the physical function score (WOMAC/KOOS) between TCE group and a control/comparison group. CI: Confidence Interval; KOOS: The Knee Injury and Osteoarthritis Outcome Score; WOMAC: The Western Ontario and McMaster Universities Arthritis Index.

The findings of the meta-analyses based on 14 included studies are presented by the forest plots in regard to outcomes of pain, stiffness, and physical function using the WOMAC or KOOS score (Figure 4, Figure 5 and Figure 6). Furthermore, all the included studies used the standardized mean difference (SMD) due to the different questionnaire scale.

#### 3.4.1. Pain

All 14 included studies totaling 815 participants reported the effects of different interventions on outcome of WOMAC/KOOS pain score. The synthesized data indicated that the TCE group had significantly alleviated pain as compared with a control/comparison group (SMD = −0.61, 95% CI: −0.86 to −0.37, *p* < 0.001), and there was a substantial heterogeneity for this synthesized outcome (I^2^ = 64%). Therefore, these studies were combined using the random-effects model (Figure 4).

#### 3.4.2. Stiffness

A total of 13 studies involving 708 participants reported the effects of different interventions on outcome of WOMAC/KOOS stiffness score [33,34,35,36,37,48,49,51,52,53,54,55,56]. The synthesized data indicated that the TCE group had effectively relieved stiffness as compared with a control/comparison group (SMD = −0.75, 95% CI: −1.09 to −0.41, *p* < 0.001), and there was a high heterogeneity for this synthesized outcome (I^2^ = 77%). Therefore, these studies were combined using the random-effects model (Figure 5).

#### 3.4.3. Physical Function

There were 13 studies involving 772 participants that reported the effects of different interventions on outcome of WOMAC/KOOS physical function score [33,34,35,36,37,48,49,50,51,52,53,55,56], The synthesized data indicated that the TCE group benefited far more for improving physical function than a control/comparison group (SMD = −0.67, 95% CI: −0.82 to −0.53, *p* < 0.001), and there was a moderate heterogeneity for this synthesized outcome (I^2^ = 34%). Therefore, these studies were combined using the fix-effects model (Figure 6).

### 3.5. Evaluation of Publication Bias

**Figure 7:**
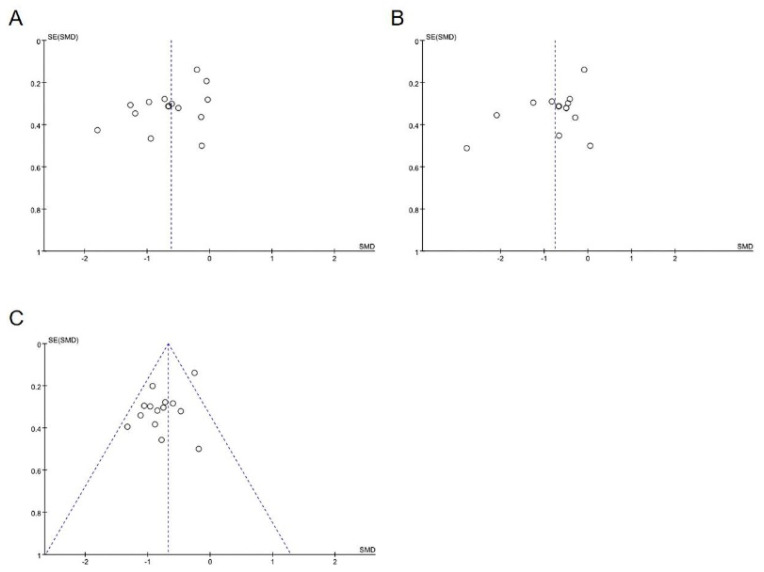
Funnel plots showing a possible publication bias favoring the positive studies on outcomes of the WOMAC/KOOS score. (**A**) Funnel plots of the WOMAC/KOOS pain score. (**B**) Funnel plots of the WOMAC/KOOS stiffness score. (**C**) Funnel plots of the WOMAC/KOOS physical function score.

The publication bias of outcome was evaluated using funnel plots based on 14 studies. The funnel plots of the WOMAC/KOOS pain score, WOMAC/KOOS stiffness score, and WOMAC/KOOS physical function score suggested a possible publication bias in small trials, the Egger’s test (*P* = 0.031; 0.028; 0.043) of DCR demonstrated that there was a publication bias. Funnel plots showing a possible publication bias favored the positive studies on outcomes of the WOMAC/KOOS score (Figure 7). It is possible that either their included studies were limited in number, or the negative outcomes did not get published.

### 3.6. Sensitivity Analysis and Subgroup Meta-Analyses

#### 3.6.1. Sensitivity Analysis

Given the high level of heterogeneity and risk of bias, sensitivity analysis was conducted to identify the robustness of the synthesized outcome. It was performed through removing each individual study. After removing 2 studies [50,52] in pain and 2 studies [34,52] in stiffness respectively, statistical heterogeneity of this synthesized outcome showed dramatically decrease in pain (SMD = −0.52, 95% CI: −0.67 to −0.37, I^2^ = 49%) and in stiffness (SMD = −0.43, 95% CI: −0.59 to −0.28, I^2^ = 39%). The reason of high heterogeneity may due to different assessment scale (WOMAC and KOOS).

#### 3.6.2. Subgroup Meta-Analyses

Subgroup analyses were performed based on the outcomes of 14 studies, which included exercise type, geographical location, duration time, sample size and control group type. All relevant data of subgroup meta-analyses are shown in Table 2 and the supplementary material (Figures S1–S5).

As shown in the table, we found that except the outcomes of pain in Baduanjin exercise (SMD = −0.61, 95% CI: −1.28 to 0.06, *p* = 0.07), the outcomes of stiffness in non-Asian populations (SMD = −0.43, 95% CI −0.90 to 0.04, *p* = 0.08) and the outcomes of symptoms in participant ≤ 30 (SMD = −0.43, 95% CI: −0.90 to 0.04, *p* = 0.08), the rest of the subgroup analyses indicated significant improvements respectively.

## 4. Discussion

The knee joint, which consists of osseous structures (distal femur, proximal tibia, and patella), cartilages (meniscus and hyaline cartilages), ligaments, and a synovial membrane, is the largest and most complex synovial joint in the human body [57]. Frequent and stressful use of the knee joint may cause serious pain associated with more severe diseases including KOA [58].

KOA is a degenerative and progressive knee-joint disease with multifactorial etiologies that have been attributed to advancing age, over-weight, female gender, genetic predisposition, trauma, mechanical forces, inflammation, biochemical reactions, and metabolic derangements [57,59,60]. The pathophysiology of KOA is characterized by degradation and destruction of cartilage (articular cartilage and subchondral bone) and meniscus, osteophyte formation, bone remodeling, peri-articular muscles weakness, laxity of ligaments and joints, and synovial inflammation. The symptoms of KOA comprise pain, stiffness, swelling, limitation movement, and function impairment of the knee joint [57,59]. These may eventually affect the whole joint and eventually lead to disability [4]. Meanwhile, KOA entails chronic inflammation and structural damage unamendable to restoration. Therefore, therapeutic regimens have aimed to improve the symptoms of KOA [57].

Currently, an intra-articular injection and cyclooxygenase inhibitors are widely used in the clinical treatment of KOA, such as extended-release triamcinolone acetonide, corticoids (CS), hyaluronic acid (HA), autologous conditioned serum (ACS), platelet-rich plasma (PRP), mesenchymal stem cell (MSC), non-steroidal anti-inflammatory drugs (NSAIDs), and acetaminophen [57,59]. The safety of pharmacological therapies is still a controversy: Patients with KOA are likely to suffer from mild to moderate adverse effects and financial burden [61,62]. Non-pharmacological therapies comprise Tai Chi and other kinds of TCE, yoga, aquatic therapies, and weight loss [15]. Additionally, the majority of studies confirmed that non-pharmacological therapies are safe and highly effective [15,63,64]. Hence, they should be administered as the primary treatment option for management of KOA symptoms [57].

Our findings showed significant improvements in pain, stiffness, and physical function with the TCE, which is consistent with results of the first meta-analysis of TCE for KOA performed in 2017 [18]. However, this former meta-analysis has included two ineligible studies, one of them included participants who also had hip osteoarthritis [65], and another study that was retracted due to poor quality [66]. Our meta-analysis conducted more subgroup analyses and included more studies (14 RCTs) and more participants (815), while the previous meta-analysis included only eight studies involving 375 participants.

Our meta-analysis demonstrated that TCE significantly alleviated pain (SMD = −0.61, 95% CI: −0.86 to −0.37, *p* < 0.001), relieved stiffness (SMD = −0.75, 95% CI: −1.09 to −0.41, *p* < 0.001), and improved physical function (SMD = −0.67, 95% CI: −0.82 to −0.53, *p* < 0.001). These benefits are consistent with the results of individual included RCTs [33,34,35,36,37,48,49,50,51,52,53,54,55,56]. For different exercise types, subgroup analyses indicated there was no statistically significant difference between Tai Chi exercise and Baduanjin exercise in WOMAC/KOOS score. Compared with a control/comparison group, Tai Chi exercise and Baduanjin exercise showed a significant improvement in stiffness score and physical function score. However, in terms of pain, Tai Chi exercise achieved a significant improvement in reducing pain (*p* < 0.0001), while Baduanjin exercise showed no significant difference (*p* = 0.07). This finding may be due to insufficient number of RCTs for the Baduanjin exercise. Therefore, further RCTs are required to investigate the effectiveness of Baduanjin exercise in treating symptoms of KOA.

In addition, several RCTs have evaluated the effectiveness of TCE to other outcomes for patients with KOA. A double-blind randomized clinical trial involving 46 patients demonstrated that Tai Chi could effectively improve gait velocity, step length, and initial contact angle and maximal angle of flexed knees during the stance phase of walking [35]. Another RCT indicated that the Tai Chi group achieved better health behavior after 12 weeks, specifically on a diet behavior and stress management [54]. Meanwhile, a randomized, single-blind, four-arm, clinical trial involving 140 patients demonstrated that the Tai Chi and Baduanjin exercise could significantly relieve pain by simultaneously modulating the resting-state functional connectivity of the descending opioidergic pathway and the dopamine reward/motivation system [51]. There is a single-blind RCT indicating that the Tai Chi group is beneficial for balance and strengthening of the abdominal muscle [53]. Other single-blind RCTs indicated that the Tai Chi/Baduanjin exercise significantly contributed to proprioception and postural stability of the knee joint [34], quadriceps strength [48], self-efficacy [56], depression [33,56], and health-related quality of life [33,50,56]. Overall, although the underlying mechanisms of TCE are unknown, it may be an effective treatment regimen for physical and psychological health of patients with KOA.

There are methodological limitations that should be noted. First, all the included RCTs were published in the English language only—this may lead to a language bias, as well as a publication bias that positive results were much more likely reported in journals. However, the English articles are of a much higher quality than the Chinese articles, so only high-quality English articles were included in this study [67,68,69,70]. In addition, a publication bias might have existed in the included RCTs since positive trials are more likely to be published than negative results. Second, most of the included RCTs had significant flaws on methodological characteristics. About 70% of the included RCTs’ sample size were less than 30, and some of them lacked concealment of allocation and bias of subjective and report, which could limit the strength of our positive results. Third, the TCE intervention protocol varied widely with respect to the exercise type (Tai Chi and Baduanjin), duration time (from 4 to 24 weeks), frequency (two to twelve times per week), and control group that received different interventions. To a certain degree, it is difficult for us to make an optimal training recommendation for health-care professionals and the general public on which type of exercise should be selected, how long the exercise should last, and how frequent the exercise should be. Finally, in some included RCTs, TCE was not mono-therapy to patients but mixed with medicines or usual care, which is hard to confirm whether the positive results were contributed by TCE alone or a synergetic intervention effect (i.e., a combination of TCE and medicines or both TCE and usual care). However, it must be admitted that the improvement of symptoms was achieved in patients with KOA during the TCE intervention period.

## 5. Conclusions

This meta-analysis suggests that TCE may be an effective intervention approach for patients with symptoms of KOA including pain, stiffness, and physical function. Moreover, there are publication and language biases that may overestimate the effectiveness of the TCE intervention. However, without the risk of significant adverse events, clinicians can still consider TCE as an adjuvant therapy incorporating it into their first-line rehabilitation regime for patients with KOA. In addition, given the methodological limitations of the included RCTs in this meta-analysis, which might have impacted the interpretation of these findings, more high-quality RCTs with a large sample size and long-term intervention are required to further confirm the effectiveness and underlying mechanisms of TCE for treating KOA.

## Figures and Tables

**Table 1:** Characteristics of data extracted from the included studies.

Reference	Study Location	Participant Characteristics			Intervention Protocol			Outcomes Measure	Adverse Effects
		Patients Diagnostic Criteria	Sample Size (IG/CG)	Mean Age or Age Range	Intervention Group	Control Group	Duration Time		
AN et al., 2008 [48]	Shanghai, China	ACR	11/10	IG:65.4 ± 8.2 CG:64.6 ± 6.7	Baduanjin 5 × 30 min/week	None	8 weeks	WOMAC a. Pain b. Stiffness c. Physical function	No adverse event
Brisme’e et al., 2007 [49]	Texas, USA	ARA	18/13	IG:70.8 ± 9.8 CG:68.8 ± 8.9	Tai Chi 3 × 40 min/week	Attention control pre-6 weeks 3 × 40 min/week post-6 weeks no attend activity	12 weeks	WOMAC a. Pain b. Stiffness c. Physical function	No adverse event
Lee et al., 2009 [36]	Hwaseong, Korea	KL scale	29/15	IG:70.2 ± 4.8 CG:66.9 ± 6.0	Tai Chi 2 × 60 min/week	None	8 weeks	WOMAC a. Pain b. Stiffness c. Physical function	No adverse event
Li et al., 2019 [50]	Jining, China	Radiographic evidence	54/53	IG:69.6 ± 4.3 CG:68.5 ± 3.5	Tai Chi 5 × 45 min/week	Traditional physical exercises 5 × 45 min/week	12 weeks	WOMAC a. Pain b. Physical function	No adverse event
Liu et al., 2019 [51]	Fujian, China	ARA	1.28/24 2.29/24	IG1:40–70 IG2:40–68 CG:40–70	1: Tai Chi 5 × 60 min/week 2: Baduanjin 5 × 60 min/week	Healthy education 1 × 60 min/week	12 weeks	KOOS a. Pain b. Symptoms c. Daily living	No adverse event
Michael et al., 2013 [37]	Knoxville, USA	ACR	12/6	IG:68.1 ± 5.3 CG:70.5 ± 5.0	Tai Chi 5 × 60 min/week	Not intervention	10 weeks	WOMAC a. Pain b. Stiffness c. Physical function	No adverse event
Mona et al., 2018 [52]	Tehran, Iran	KL scale	16/16	IG:55.25 ± 5.72 CG:56.06 ± 6.13	Tai Chi 12 × 45 min/4 week and Routin physiotherapy 12 × 20 min/4 week	Routine physiotherapy 12 × 20 min/4 week	4 weeks	KOOS a. Pain b. Symptoms c. Daily living	No adverse event
Song et al., 2003 [53]	Seoul, Korea	ACR	22/21	IG:64.8 ± 6.0 CG:62.5 ± 5.6	Tai Chi pre-2 weeks 3 × 20 min/week post-10 weeks 3 × 20 min/week	None	12 weeks	WOMAC a. Pain b. Stiffness c. Physical function	No adverse event
Song et al., 2007 [54]	Seoul, Korea	Radiographic evidence	22/21	IG:64.8 ± 6.0 CG:62.5 ± 5.6	Tai Chi pre-2 weeks 3 × 60 min/week post-10 weeks 3 × 60 min/week	None	12 weeks	WOMAC a. Pain b. Stiffness	No adverse event
Tsai et al., 2012 [55]	Arkansas, USA	health care provider	28/27	IG:78.89 ± 6.91 CG:78.93 ± 8.30	Tai Chi 3 × 20–40 min/week	Attention control 3 × 20–40 min/week	20 weeks	WOMAC a. Pain b. Stiffness c. Physical function	No adverse event
Wang et al., 2009 [56]	Boston, USA	ACR	20/20	IG:63 ± 8.1 CG:68 ± 7.0	Tai Chi 2 × 60 min/week	Attention control 2 × 60 min/week	12 weeks	WOMAC a. Pain b. Stiffness c. Physical function	One participant in the Tai Chi group reported an increase in knee pain
Wang et al., 2016 [33]	Boston, USA	ACR	106/98	IG:60.3 ± 10.5 CG:60.1 ± 10.5	Tai Chi 2 × 60 min/week	Physical therapy pre-6 weeks 2 × 30 min/week post-6 weeks 4 × 30 min/week	12 weeks	WOMAC a. Pain b. Stiffness c. Physical function	No adverse event
Ye et al., 2020 [34]	Fujian, China	ACR	25/25	IG:64.48 ± 7.81 CG:63.08 ± 3.65	Baduanjin 3 × 40 min/week	None	12 weeks	WOMAC a. Pain b. Stiffness c. Physical function	No adverse event
Zhu et al., 2016 [35]	Shanghai, China	ACR	23/23	IG:64.61 ± 3.40 CG:64.53 ± 3.43	Tai Chi 3 × 60 min/week	Healthy education 1 × 60 min/week	24 weeks	WOMAC a. Pain b. Stiffness c. Physical function	No adverse event

Abbreviations: ACR: American College of Rheumatology; ARA: American Rheumatism Association; CG: Control Group; IG: Intervention Group; KL: Kellgren–Lawrence Scale; KOOS: The Knee injury and Osteoarthritis Outcome Score; WOMAC: The Western Ontario and McMaster Universities Osteoarthritis Index.

**Table 2:** Subgroup meta-analyses.

Subgroups	Outcomes	No. of Studies	No. of Sample Size	SMD (95% CI)	Statistical Method	*p*-Value for Heterogeneity
Exercise type						
Tai Chi	Pain	12	715	−0.62 [−0.90, −0.34]	Random effect	<0.0001 **
	Stiffness	11	608	−0.67 [−1.02, −0.32]	Random effect	0.0002 **
	Physical function	11	672	−0.62 [−0.78, −0.46]	Fix effect	<0.00001 **
Baduanjin	Pain	3	124	−0.61 [−1.28, 0.06]	Random effect	0.07
	Stiffness	3	124	−1.05 [−2.13, 0.02]	Random effect	0.05 *
	Physical function	3	124	−0.97 [−1.34, −0.59]	Fix effect	<0.00001 **
Geographical location						
Asian populations	Pain	9	467	−0.69 [−1.02, −0.36]	Random effect	<0.0001 *
	Stiffness	8	360	−0.94 [−1.38, −0.50]	Random effect	<0.0001 **
	Physical function	8	424	−0.85 [−1.04, −0.65]	Fix effect	<0.00001 **
Non−Asian populations	Pain	5	348	−0.47 [−0.87, −0.08]	Random effect	0.02 *
	Stiffness	5	348	−0.43 [−0.90, 0.04]	Random effect	0.08
	Physical function	5	348	−0.60 [−0.97, −0.22]	Random effect	0.002 **
Duration time						
8 weeks	Pain	2	65	−0.64 [−1.16, −0.12]	Fix effect	0.02 *
	Stiffness	2	65	−0.55 [−1.06, −0.03]	Fix effect	0.04 *
	Physical function	2	65	−0.57 [−1.09, −0.05]	Fix effect	0.03 *
12 weeks	Pain	8	599	−0.54 [−0.85, −0.23]	Random effect	0.0007 **
	Stiffness	7	492	−0.66 [−1.06, −0.26]	Random effect	0.001 **
	Physical function	7	556	−0.78 [−1.05, −0.50]	Random effect	<0.00001 **
Sample size						
n ≥ 30	Pain	12	776	−0.62 [−0.89, −0.36]	Random effect	<0.00001 **
	Stiffness	11	669	−0.81 [−1.19, −0.44]	Random effect	<0.0001 **
	Physical function	11	733	−0.68 [−0.83, −0.53]	Fix effect	<0.00001 **
n < 30	Pain	2	39	−0.56 [−1.23, 0.11]	Fix effect	0.1
	Stiffness	2	39	−0.34 [−1.00, 0.31]	Fix effect	0.31
	Physical function	2	39	−0.50 [−1.17, 0.16]	Fix effect	0.13
Control group type						
Active control group	Pain	8	596	−0.71 [−1.07, −0.35]	Random effect	0.0001 **
	Stiffness	7	489	−0.74 [−1.19, −0.29]	Random effect	0.001 **
	Physical function	8	596	−0.78 [−1.04, −0.52]	Random effect	<0.00001 **
Passive control group	Pain	6	219	−0.46 [−0.73, −0.18]	Fix effect	0.001 **
	Stiffness	6	219	−0.78 [−1.32, −0.23]	Random effect	0.005 **
	Physical function	5	176	−0.71 [−1.02, −0.40]	Fix effect	<0.00001 **

Abbreviations: CI = Confidence Intervals; SMD = Standardized Mean Different; * *p* < 0.05, ** *p* < 0.01, compared TCE group with control group.

## Supplementary Materials

The following are available online at https://www.mdpi.com/1660-4601/17/21/7873/s1, Figure S1: Subgroup analyses were performed based on different exercise type. (A) Tai Chi, (B) Baduanjin. Figure S2: Subgroup analyses were performed based on different geographical location. (A) Asian populations, (B) Non-Asian populations. Figure S3: Subgroup analyses were performed based on different duration time. (A) 8 weeks, (B) 12weeks. Figure S4: Subgroup analyses were performed based on different sample size. (A) No. of participants ≥ 30, (B) No. of participants <30. Figure S5: Subgroup analyses were performed based on different control group type. (A) Active control group, (B) Passive control group. Table S1: Search strategy and result of each database.

## Abbreviations

AAOS	American Academy of Orthopedic Surgeons
ACR	American College of Rheumatology classification criteria
ACS	Autologous Conditioned Serum
ARA	American Rheumatism Association
CG	Control Group
CI	Confidence Interval
HA	Hyaluronic Acid
IG	Intervention Group
KL	the Kellgren Lawrence classification
KOA	Knee Osteoarthritis
KOOS	the Knee Injury and Osteoarthritis Outcome Score
MSC	Mesenchymal Stem Cell
NSAIDs	Non-Steroidal Anti-Inflammatory Drugs
OARSI	Osteoarthritis Research Society International
PRP	Platelet-Rich Plasma
RCT	Randomized Controlled Trial
SMD	Standardized Mean Difference
TCE	TCE
WMD	Weighted Mean Difference
WOMAC	the Western Ontario and McMaster Universities Osteoarthritis Index
