# Supplementary material for: Effectiveness of Traditional Chinese Exercise for Symptoms of Knee Osteoarthritis: A Systematic Review and Meta-Analysis of Randomized Controlled Trials
Source: Int J Environ Res Public Health. 2020 Oct 27;17(21):7873. doi: 10.3390/ijerph17217873 (PMC7662219; doi:10.3390/ijerph17217873)
Supplement: Supplementary file 1 [file ijerph-17-07873-s001.pdf]

Table S1. Search strategy and result of each database.

Database	Search Strategies	Number of Articles
PubMed	(("Qigong"[MeSH] OR "Qi Gong"[Title/Abstract] OR "Chi Kung"[Title/Abstract] OR "yijinjing"[Title/Abstract] OR "baduanjin"[Title/Abstract] OR "wuqinxi"[Title/Abstract] OR "Tai Ji"[MeSH] OR "Tai-Ji"[Title/Abstract] OR "Tai Chi"[Title/Abstract] OR "Chi Tai"[Title/Abstract] OR "Tai Ji Quan"[Title/Abstract] OR "Quan Tai Ji"[Title/Abstract] OR "Ji Quan Tai"[Title/Abstract] OR "Taiji"[Title/Abstract] OR "Taijiquan"[Title/Abstract] OR "T'ai Chi"[Title/Abstract] OR "Tai Chi Chuan"[Title/Abstract] OR "traditional Chinese exercise"[Title/Abstract]) AND ("Osteoarthritis, Knee"[MeSH] OR "Knee Osteoarthritides"[Title/Abstract] OR "Knee Osteoarthritis"[Title/Abstract] OR "Osteoarthritis of Knee"[Title/Abstract] OR "Osteoarthritis of the Knee"[Title/Abstract] OR "gonitis"[Title/Abstract] OR "gonarthritis"[Title/Abstract] OR "Knee Arthritis"[Title/Abstract]) AND ("randomized controlled trial"[Title/Abstract] OR "randomization"[Title/Abstract] OR "randomized"[Title/Abstract]))	40
Web of Science	((TI=(Qigong OR Qi Gong OR Chi Kung OR yijinjing OR baduanjin OR wuqinxi OR Tai Ji OR Tai-ji OR Tai Chi OR Chi Tai OR Tai Ji Quan OR Quan Tai Ji OR Ji Quan Tai OR Taiji OR Taijiquan OR T'ai Chi OR Tai Chi Chuan OR traditional Chinese exercise) OR AB=(Qigong OR Qi Gong OR Chi Kung OR yijinjing OR baduanjin OR wuqinxi OR Tai Ji OR Tai-ji OR Tai Chi OR Chi Tai OR Tai Ji Quan OR Quan Tai Ji OR Ji Quan Tai OR Taiji OR Taijiquan OR T'ai Chi OR Tai Chi Chuan OR traditional Chinese exercise)) AND (TI=(Osteoarthritis, Knee OR Knee Osteoarthritides OR Knee Osteoarthritis OR Osteoarthritis of Knee OR Osteoarthritis of the Knee OR gonitis OR gonarthritis OR Knee Arthritis) OR AB=(Osteoarthritis, Knee OR Knee Osteoarthritides OR Knee Osteoarthritis OR Osteoarthritis of Knee OR Osteoarthritis of the Knee OR gonitis OR gonarthritis OR Knee Arthritis)) AND (TI=(randomized controlled trial OR randomization OR randomized) OR AB=(randomized controlled trial OR randomization OR randomized)))	68
Cochrane Library	(((Qigong)MeSH OR (Qi Gong):ti,ab OR (Chi Kung):ti,ab OR (yijinjing):ti,ab OR (baduanjin):ti,ab OR (wuqinxi):ti,ab OR (Tai Ji)MeSH OR (Tai-Ji):ti,ab OR (Tai Chi):ti,ab OR (Chi Tai):ti,ab OR (Tai Ji Quan):ti,ab OR (Quan Tai Ji):ti,ab OR (Ji Quan Tai):ti,ab OR (Taiji):ti,ab OR (Taijiquan):ti,ab OR (T'ai Chi):ti,ab OR (Tai Chi Chuan):ti,ab OR (traditional Chinese exercise):ti,ab) AND ((Osteoarthritis, Knee)MeSH OR (Knee Osteoarthritides):ti,ab OR (Knee Osteoarthritis):ti,ab OR (Osteoarthritis of Knee):ti,ab OR (Osteoarthritis of the Knee):ti,ab OR (knee arthritis):ti,ab OR (gonitis):ti,ab OR (gonarthritis):ti,ab) AND ((randomized controlled trial):ti,ab, OR (randomized):ti,ab, OR (randomization):ti,ab))	80

EMBASE	(("Qigong":ab,ti OR "Qi Gong":ab,ti OR "Chi Kung":ab,ti OR "yijinjing":ab,ti OR "baduanjin":ab,ti OR "wuqinxi":ab,ti OR "Tai Ji":ab,ti OR "Tai-Ji":ab,ti OR "Tai Chi":ab,ti OR "Chi Tai":ab,ti OR "Tai Ji Quan":ab,ti OR "Quan Tai Ji":ab,ti OR "Ji Quan Tai":ab,ti OR "Taiji":ab,ti OR "Taijiquan":ab,ti OR "Tai Chi Chuan":ab,ti OR "traditional Chinese exercise":ab,ti) AND ("Osteoarthritis, Knee":ab,ti OR "Knee Osteoarthritides":ab,ti OR "Knee Osteoarthritis":ab,ti OR "Osteoarthritis of Knee":ab,ti OR "Osteoarthritis of the Knee":ab,ti OR "gonitis":ab,ti OR "gonarthritis":ab,ti OR "Knee Arthritis":ab,ti) AND ("randomized controlled trial":ab,ti OR "randomization":ab,ti OR "randomized":ab,ti))	61
Reference tracing hand-searching	1. A 1-year follow-up of an experimental study of a self-management arthritis programme with an added exercise component of clients with osteoarthritis of the knee<br>2. Comparison of effects among Tai-Chi exercise, aquatic exercise, and a self-help program for patients with knee osteoarthritis<br>3. Effects of a Sun-style Tai Chi exercise on arthritic symptoms, motivation and the performance of health behaviors in women with osteoarthritis<br>4. Effects of resistance and Tai Ji training on mobility and symptoms in knee osteoarthritis patients.<br>5. Effects of Tai Chi exercise in elderly with knee osteoarthritis<br>6. Effects of Tai Chi on gait kinematics, physical function, and pain in elderly with knee osteoarthritis--a pilot study<br>7. Effects of T'ai Chi training on function and quality of life indicators in older adults with osteoarthritis<br>8. Home based exercise programme for knee pain and knee osteoarthritis: randomised controlled trial<br>9. Influence of tai chi exercise on health status for older patients with osteoarthritis.<br>10. Impact of an Arthritis Self-Management Programme with an added exercise component for osteoarthritic knee sufferers on improving pain, functional outcomes, and use of health care services: An experimental study<br>11. The effects of aquatic and traditional exercise programs on persons with knee osteoarthritis<br>12. The Effects of Routine Physiotherapy Alone and in Combination with Either Tai Chi or Closed Kinetic Chain Exercises on Knee Osteoarthritis: A Comparative Clinical Trial Study<br>13. The effects of tai chi exercise on elders with osteoarthritis: a longitudinal study<br>14. The effect of Tai Chi exercises on postural stability and control in older patients with knee osteoarthritis<br>15. The Effect of Tai Chi on Knee Osteoarthritis Pain in Cognitively Impaired Elders: Pilot Study<br>16. Teaching Tai Chi to elders with osteoarthritis pain and mild cognitive impairment	16
	Total Number of Articles	265

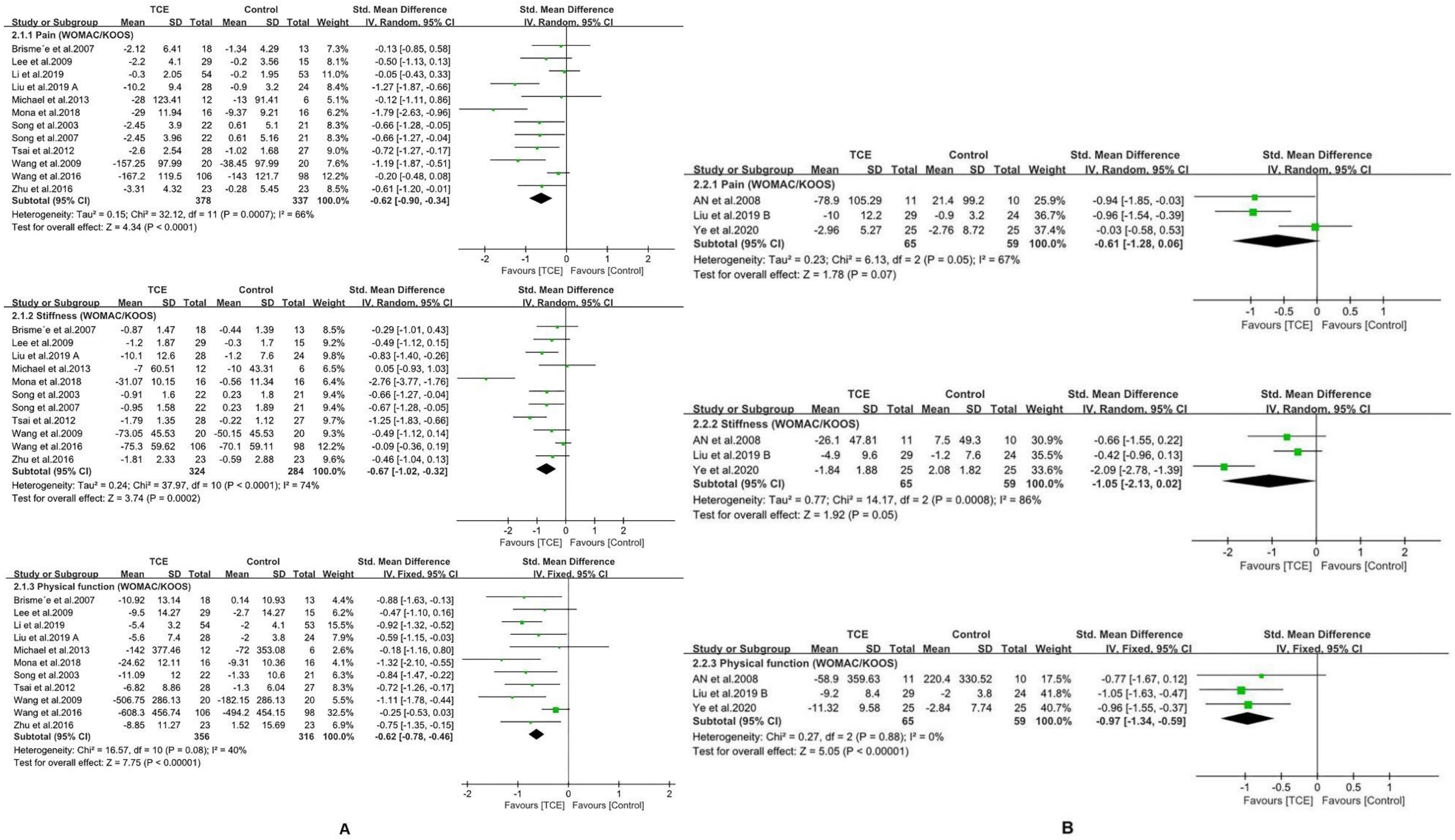

Figure S1. Subgroup analyses were performed based on different exercise type. (A) Tai Chi, (B) Baduanjin.

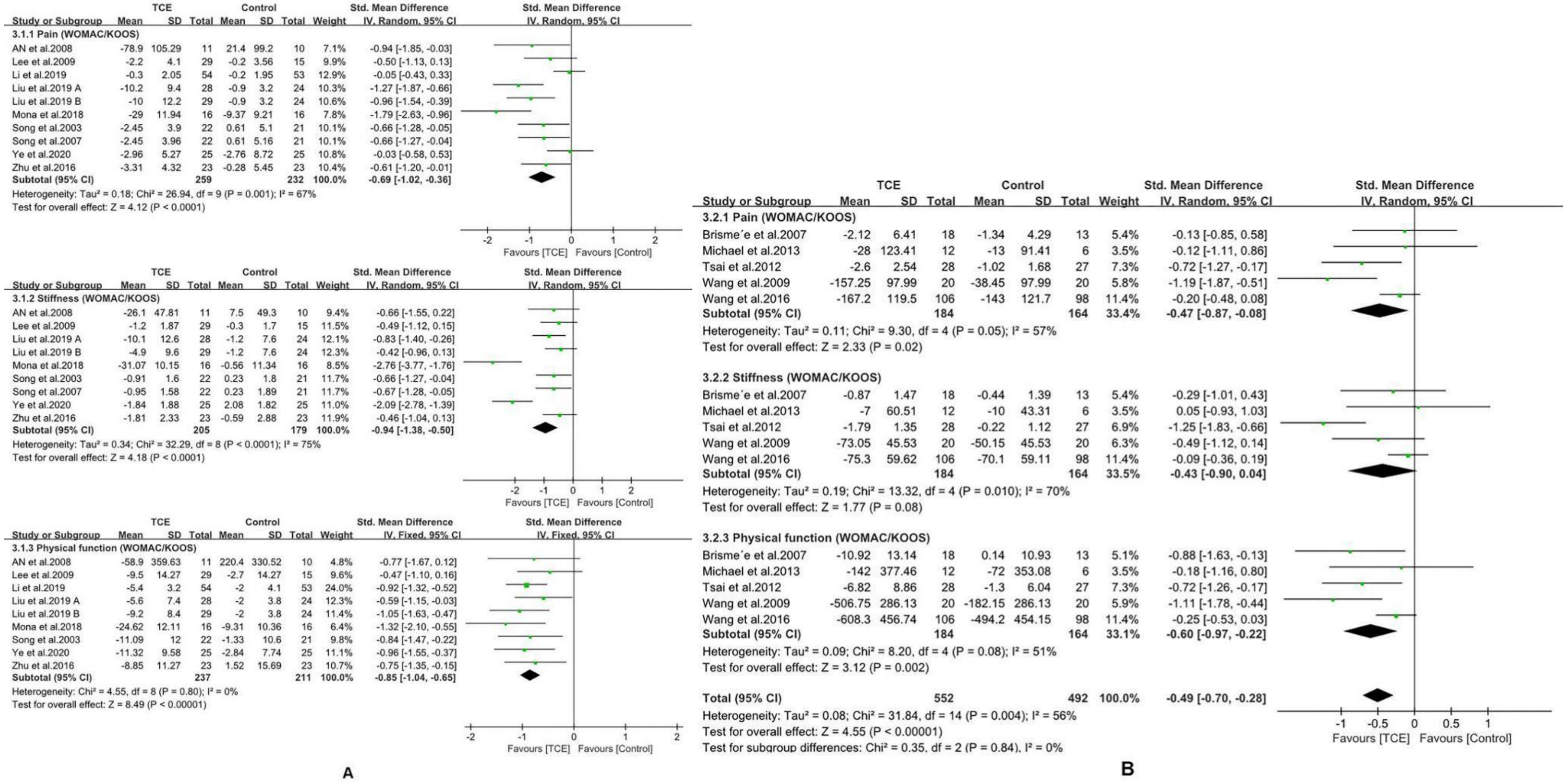

Figure S2. Subgroup analyses were performed based on different geographical location. (A) Asian populations, (B) Non-Asian populations.

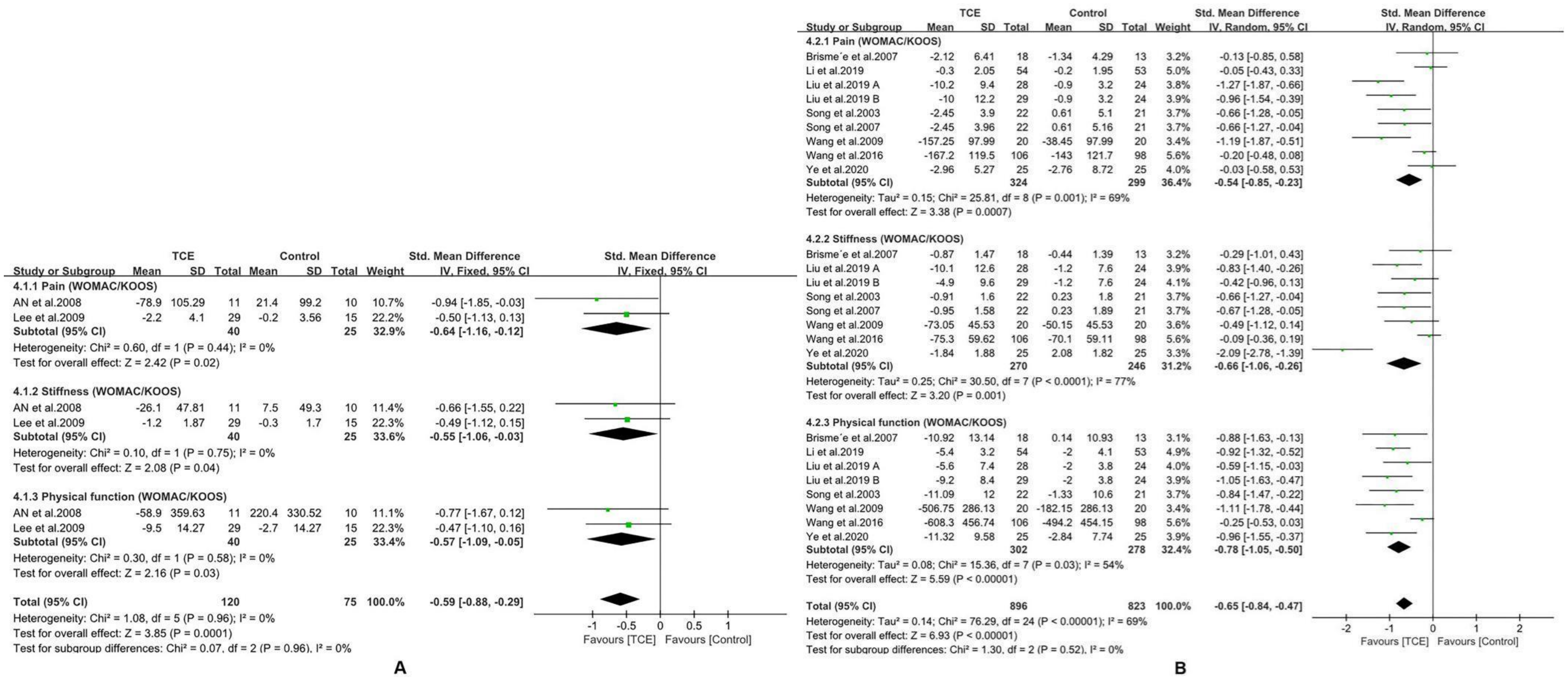

Figure S3. Subgroup analyses were performed based on different duration time. (A) 8 weeks, (B) 12weeks.

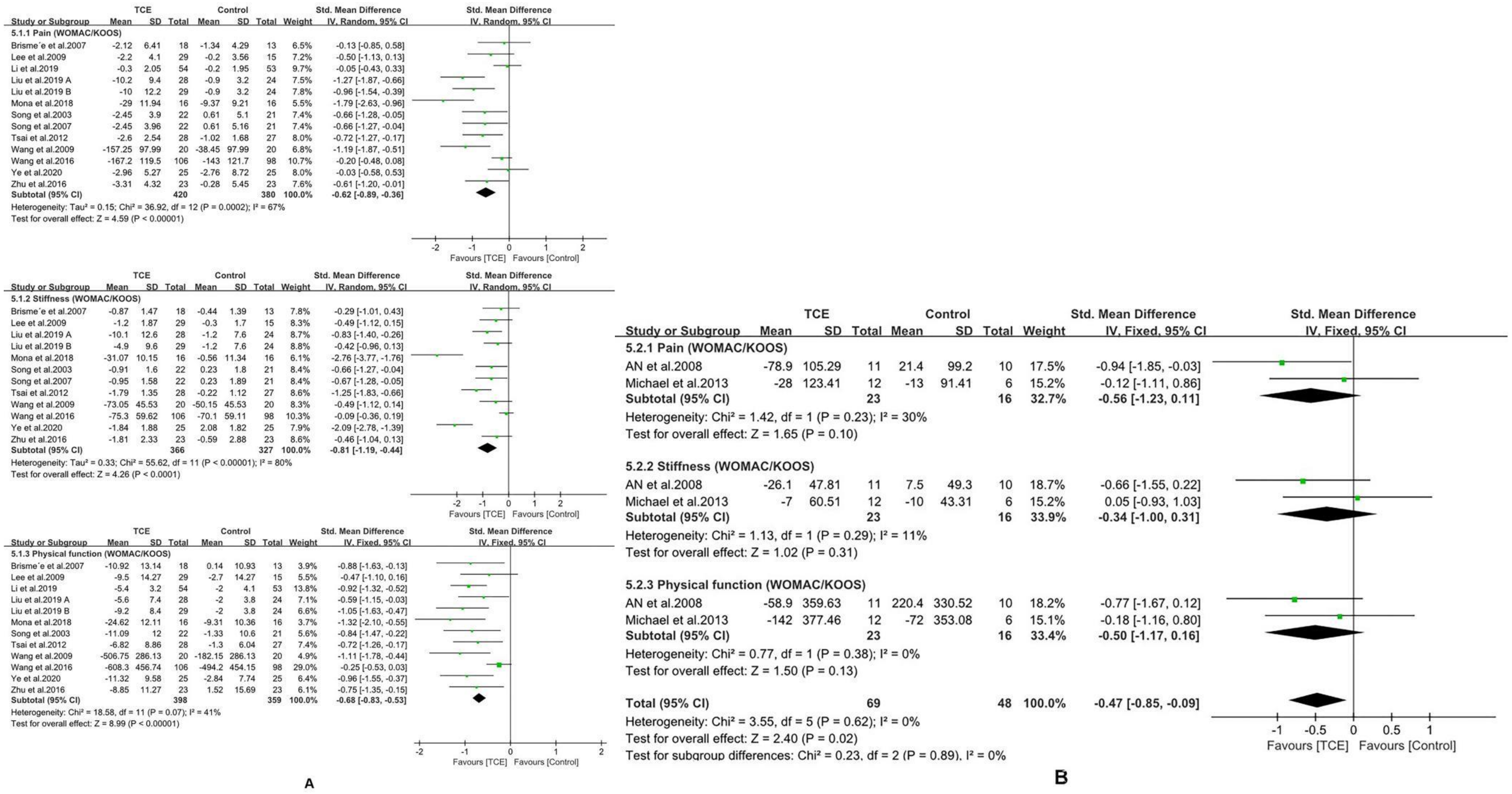

Figure S4. Subgroup analyses were performed based on different sample size. (A) No. of participants ≥ 30, (B) No. of participants <30.

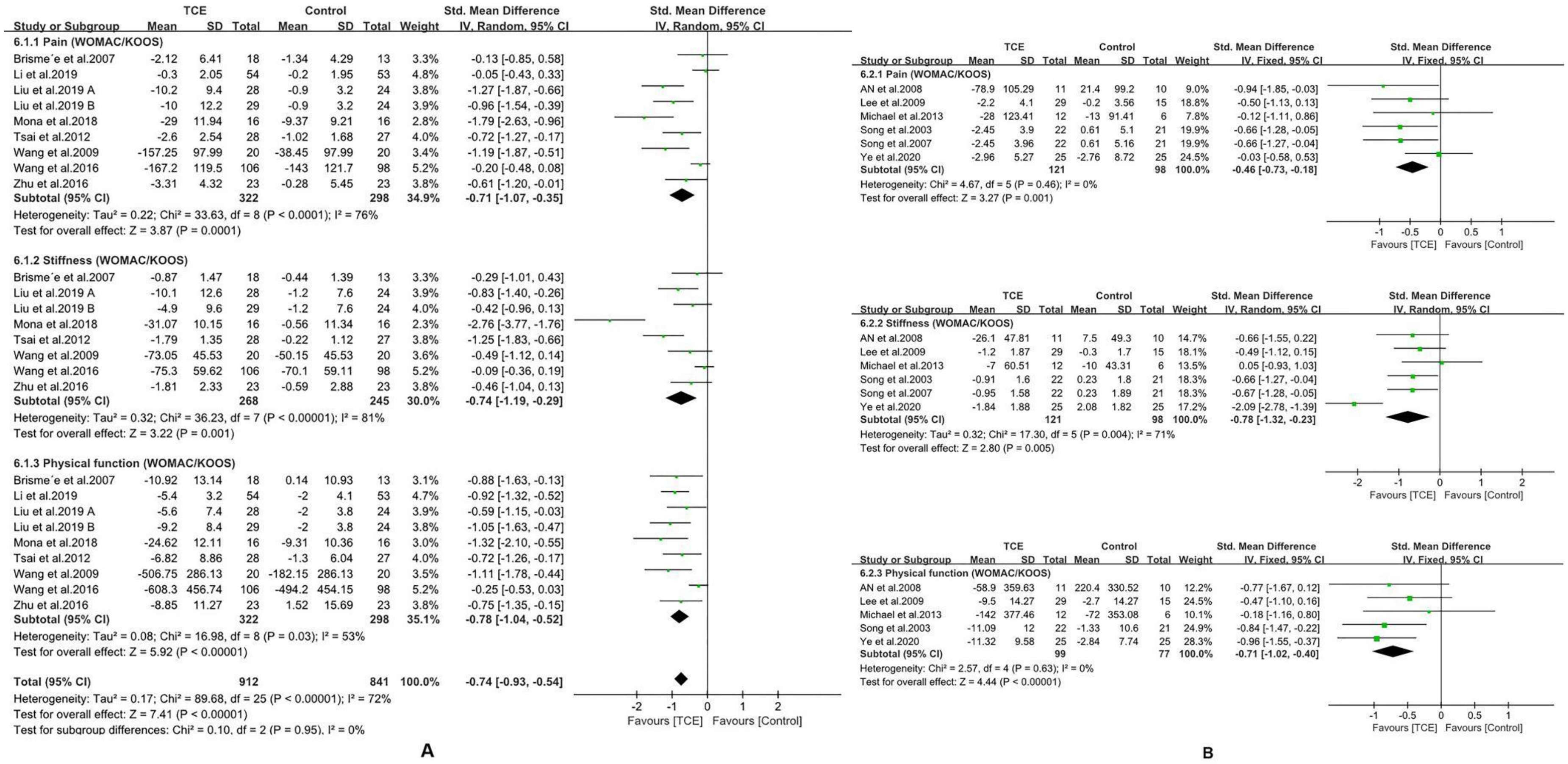

Figure S5. Subgroup analyses were performed based on different control group type. (A) Active control group, (B) Passive control group.

International Journal of
Environmental Research and Public Health
